# Delay-Selected Energy Coupling and Ablation Enhancement in Femtosecond Double-Pulse Irradiation of Aluminum

**DOI:** 10.3390/molecules31213894

**Published:** 2026-10-28

**Authors:** Feifei Wang, Jiaxuan Liu, Kai Sun, Yimo Liu, Zhe Peng, Yuntao Lang, Yueyuan Liu, Changji Pan

**Affiliations:** 1 College of Control Science and Engineering, China University of Petroleum (East China), Qingdao 266580, China; wangfeifei@upc.edu.cn (F.W.); z25050062@s.upc.edu.cn (J.L.); s25050003@s.upc.edu.cn (Y.L.); s24050004@s.upc.edu.cn (Z.P.); s24050048@s.upc.edu.cn (Y.L.); b23050001@s.upc.edu.cn (Y.L.); 2 Sinopec Petroleum Engineering Corporation, Dongying 257026, China; sunk228.osec@sinopec.com; 3 School of Mechanical Engineering and Automation, Fuzhou University, Fuzhou 350108, China

**Keywords:** femtosecond laser ablation, double-pulse irradiation, aluminum thin film, pump–probe microscopy, Fermi smearing

## Abstract

We investigate the effect of inter-pulse delay on femtosecond laser ablation of thin aluminum films using a dual-pulse setup at 800 nm with precisely controlled temporal separation. The ablation threshold, measured by an extrapolation method, first decreases and then increases with delays, reaching a minimum at ~1 ps. To reveal the underlying mechanism, time-resolved pump–probe reflection microscopy at 400 nm is employed. The transient reflectivity exhibits an ultrafast drop upon excitation, attributed to enhanced intraband absorption arising from Fermi smearing in the *sp*-band. At delays near 1 ps, a clear inflection in the reflectivity transient indicates the onset of surface thermal expansion and density reduction, which impedes efficient energy deposition into deeper material by the second pulse, thus lowering the ablation threshold. These results provide direct time-resolved evidence linking transient thermomechanical responses to delayed ablation threshold in femtosecond laser processing of aluminum.

## 1. Introduction

Ultrashort laser pulses are widely used for precise micro- and nanoscale processing of thin metallic films, with applications in plasmonic sensing, laser-induced forward transfer, and additive manufacturing [1,2,3,4]. Because the pulse duration is much shorter than the electron–phonon relaxation time in metals, laser energy is first deposited into the electronic subsystem, decoupling electronic excitation from subsequent thermal processes such as melting, thermal expansion, and ablation [5,6]. This temporal separation has motivated extensive experimental and theoretical work on the individual stages of laser–metal interaction, from prompt photoexcitation to spallation and phase explosion [7,8,9].

Aluminum is a prototypical *sp*-band metal whose optical response in the visible and near-infrared is governed by intraband (Drude) transitions. At 800 nm, the photon energy (~1.55 eV) is well below the interband transition threshold of Al, so laser energy is absorbed primarily by the free electron gas [10,11]. This raises the electron temperature to several thousand Kelvin within ~100 fs and produces pronounced Fermi smearing, i.e., a broadening of the occupation probability around the Fermi level that creates transient unoccupied states below E_F_ and occupied states above it [12,13]. Such non-equilibrium conditions enable strong intraband absorption of a 400 nm probe pulse, giving rise to an ultrafast reflectivity drop that serves as a fingerprint of the transient electronic state [14].

Double-pulse irradiation provides a route to modulate ablation by allowing the second pulse to interact with the transient state prepared by the first [15,16,17,18]. Previous theoretical study predicted that the tensile pressure wave launched by the first pulse could interfere with the compressive wave generated by the second, thereby suppressing material removal [19]. Subsequent experiments on aluminum films and bulk aluminum revealed a substantial increase in the double-pulse ablation threshold at delays of tens to hundreds of picoseconds, effectively preventing ablation at fluences that would otherwise cause material removal [20,21]. This behavior has been linked to stress relaxation dynamics in the molten layer and to screening of the incident beam by ablation products, as evidenced by time-resolved optical measurements combined with atomistic–continuum modeling [20,22,23]. In femtosecond laser ablation of metals, the rapidly heated electron gas can induce thermionic emission and electron–ion decoupling, forming a transient plasma layer above the target [24,25]. This plasma may absorb or scatter part of the incident pulse, modifying the effective energy deposition [26]. In double-pulse irradiation, the plasma density evolves with the inter-pulse delay, so its interaction with the second pulse is delay-dependent [19,20].

In contrast, the short-delay regime (sub-picosecond to a few picoseconds) remains less explored. Previous study showed that melting in aluminum occurs within a few picoseconds after excitation [27], and that the transient reflectivity evolves through stages corresponding to electronic heating, melting, thermal expansion, and acoustic relaxation, with characteristic timescales that depend on the deposited fluence [28,29]. However, a quantitative connection between the transient reflectivity dynamics at these short delays and the resulting ablation threshold has not yet been established, especially for thin aluminum films where thermal and acoustic relaxation are faster than in bulk material [21,30,31].

In this work, we study how the inter-pulse delay affects femtosecond laser ablation of thin aluminum films with precisely controlled temporal separation. The ablation threshold, obtained by the extrapolation method, shows a non-monotonic dependence on delay: it first decreases and then increases, with a minimum near 1 ps. Time-resolved pump–probe reflection microscopy at 400 nm indicates that the ultrafast reflectivity drop is dominated by intraband absorption enhanced by Fermi smearing in the *sp*-band, while the inflection near 1 ps marks the onset of surface thermal expansion and density reduction. This transient low-density surface layer hinders efficient energy deposition from the second pulse into the deeper material, leading to a lower ablation threshold. These results provide time-resolved optical evidence that correlates the transient thermomechanical response with the delayed ablation threshold in femtosecond laser processing of aluminum, suggesting a route for all-optical control of ultrafast laser ablation.

## 2. Results

### 2.1. Experimental Configuration

**Figure 1:**
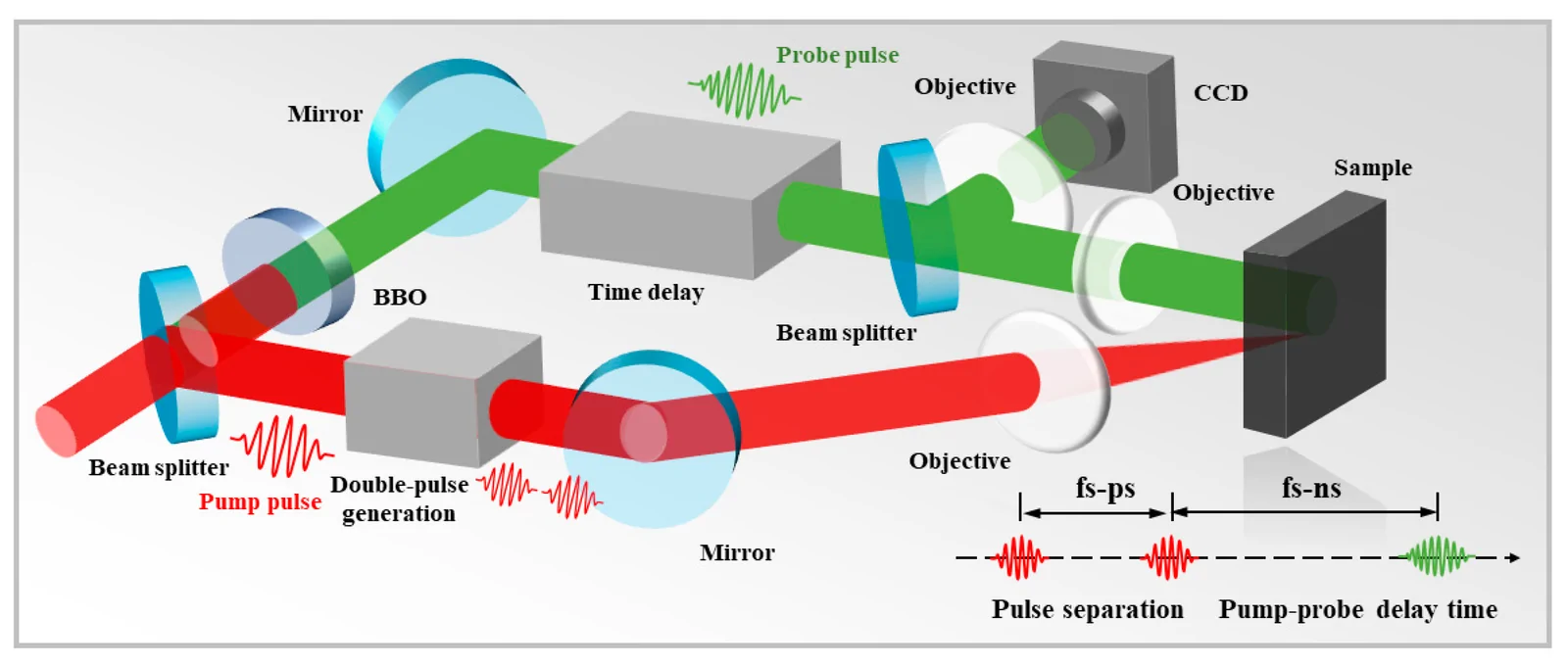
Schematic of the experimental setup. A Michelson interferometer generates two collinear 800 nm pump pulses (red line) with a tunable inter-pulse delay, and a 400 nm probe pulse (green line) is used to record the transient reflectivity through a microscope objective and a CCD camera. The inclined-incidence geometry (45°) is used for the time-resolved pump–probe reflectivity measurements, and the ablation threshold measurements were performed separately under normal incidence. The lower-right inset defines the pulse separation (fs–ps) and the pump–probe delay time (fs–ns).

The experimental setup is schematically shown in Figure 1. A Michelson interferometer configuration generates two collinear pump pulses with a central wavelength of 800 nm and a duration of ~50 fs, with precisely tunable inter-pulse delay. For the time-resolved pump–probe reflectivity measurements, the pump pulses were incident on the sample at an angle of 45°. In contrast, the ablation threshold measurements were performed separately under normal incidence. The inter-pulse delay is adjusted by translating one arm of the interferometer with a motorized one-dimensional linear translation stage, corresponding to a temporal resolution of approximately 6.7 fs per micrometer of path difference. The zero delay between the two pump pulses was determined by monitoring the spatial interference fringes formed when the two pulses overlap in time, with zero delay defined as the position where the fringe contrast reaches its maximum. To eliminate polarization-induced artifacts, a wire-grid polarizer is inserted after the delay line to ensure that both pulses share identical linear polarization. A separate 400 nm probe pulse, derived from second-harmonic generation in a BBO crystal, is focused onto the same sample spot, and its reflection is collected by a microscope objective and recorded by a CCD camera. This inclined-incidence configuration is used exclusively for the time-resolved pump–probe reflectivity measurements with sub-picosecond temporal resolution, whereas the ablation threshold measurements by the extrapolation method were performed separately under normal incidence. The lower-right inset of Figure 1 illustrates the definitions of pulse separation (fs–ps) and pump–probe delay time (fs–ns).

### 2.2. Delay-Dependent Ablation Morphology

**Figure 2:**
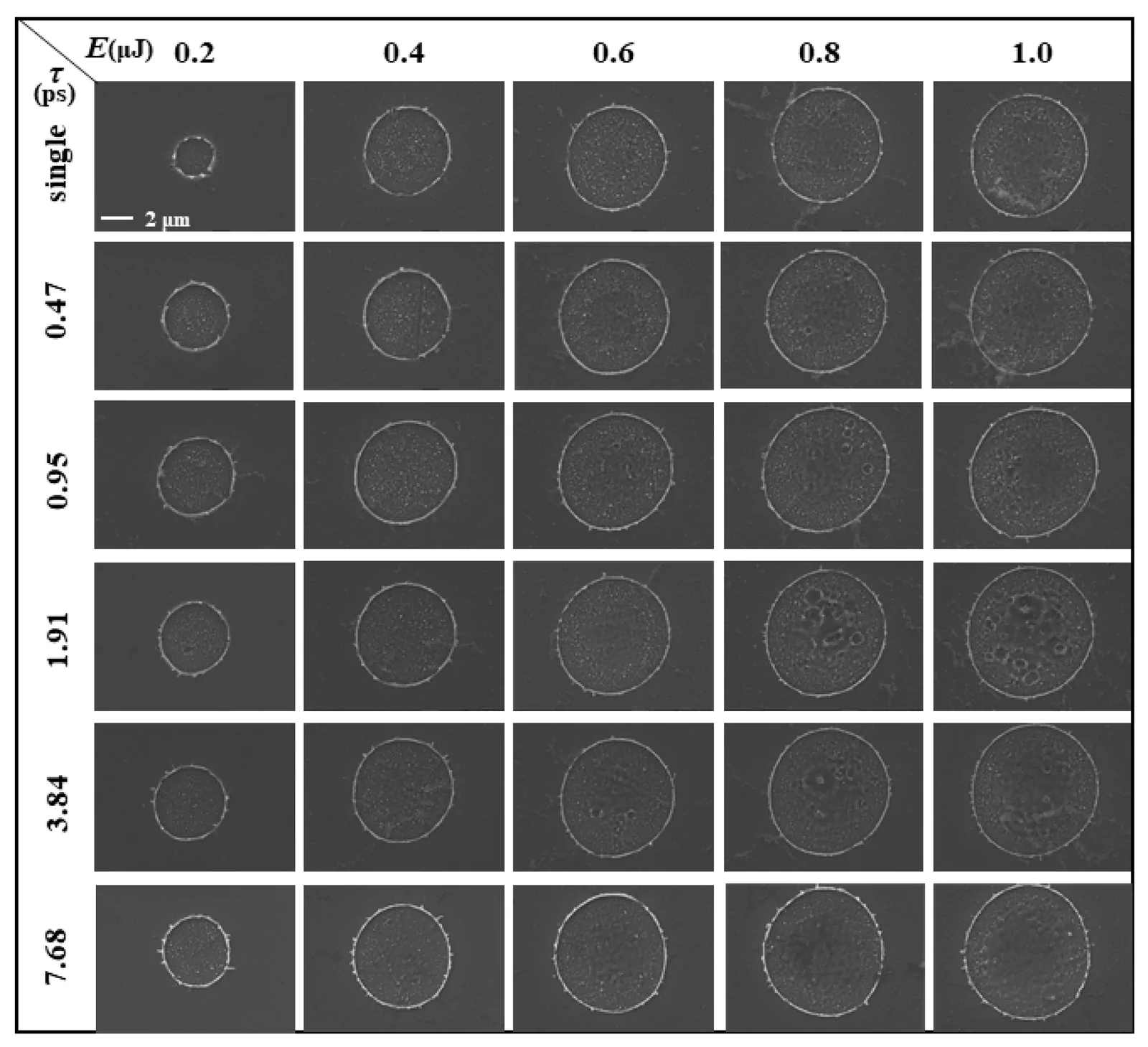
Top-view SEM images of femtosecond laser ablation craters on aluminum films as a function of inter-pulse delay (rows: single pulse, 0.47, 0.95, 1.91, 3.84, and 7.68 ps) and incident pulse energy (columns: 0.2, 0.4, 0.6, 0.8, and 1.0 μJ). The scale bar is 2 μm. Crater size and contrast decrease markedly at delays beyond ~4 ps, indicating suppression of ablation.

Figure 2 presents a matrix of top-view SEM images of ablation craters formed on aluminum films at various inter-pulse delays and incident pulse energies. For the single-pulse case, the craters are notably small, particularly at the lowest pulse energy of 0.2 μJ, where only a faint, shallow pit is visible. This indicates a reduced material removal efficiency for the single-pulse case under these conditions. At short delays (sub-picosecond to a few picoseconds), the craters are large and well-defined, indicating efficient material removal. Specifically, at delays of 0.47 ps and 0.95 ps, the craters exhibit the largest diameters and sharpest edges, indicating the highest material removal efficiency. As the delay increases to 1.91 ps, the crater edges become blurred and the central region shows inhomogeneous ablation morphology, suggesting a transition from efficient removal to suppressed ablation. This trend becomes more pronounced at delays of 3.84–7.68 ps, where the craters decrease substantially in diameter, indicating strong suppression of ablation. These observations qualitatively establish that the ablation yield is strongly modulated by the temporal separation between the two pulses.

### 2.3. Ablation Threshold Analysis

**Figure 3:**
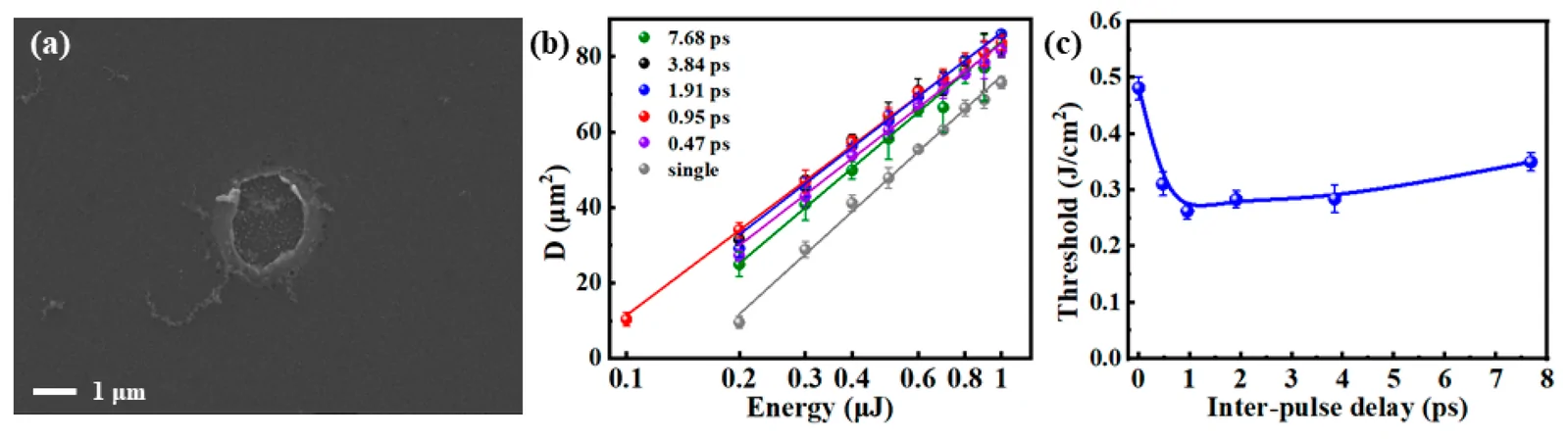
Ablation threshold analysis. (**a**) Representative SEM image of a crater produced at an inter-pulse delay of 0.95 ps and pulse energy of 0.1 μJ. (**b**) Squared crater diameter *D*^2^ versus incident pulse energy; the ablation threshold fluence is extracted by linear extrapolation to *D*^2^ = 0. (**c**) Ablation threshold fluence F as a function of inter-pulse delay *τ*. The threshold first decreases, reaches a minimum near *τ* = 1 ps, and then rises gradually at delays of 3.84–7.68 ps.

To quantify this effect, Figure 3 shows the ablation threshold analysis. Figure 3a displays a representative SEM image of a crater produced at a delay of 0.95 ps and a pulse energy of 0.1 μJ. It is worth noting that this is the only delay at which a well-defined crater forms under this pulse energy; for other inter-pulse delays, no discernible ablation occurs at the same fluence, and thus no comparable crater images are available. This contrast provides direct morphological evidence that the ablation threshold is substantially lowered at the ~1 ps delay.

The ablation threshold fluence *F*th was determined by the extrapolation method. For a Gaussian beam, the squared crater diameter *D*^2^ depends linearly on the logarithm of the incident pulse energy *E*:

(1) $$D^{2}=2w_{0}^{2}\ln\left(\frac{2E}{\pi w_{0}^{2}F_{\mathrm{th}}}\right)$$

where *w*_0_ ≈ 3.6 μm is the beam waist radius for the normal-incidence ablation threshold measurements, determined from the slope of the *D*^2^ versus ln*E* fit. By extrapolating the linear fit to *D*^2^ = 0, the threshold energy *E*th was obtained, and the threshold fluence was calculated as $F_{\mathrm{th}}=2E_{\mathrm{th}}/\pi w_{0}^{2}$. Equation (1) assumes that ablation occurs wherever the local fluence exceeds a spatially sharp threshold and that the beam profile is Gaussian. Under these conditions, the squared crater diameter remains linear in ln E irrespective of whether the removal mechanism is governed by optical penetration, electron heat penetration, or plasma-mediated ablation. The linearity observed in Figure 3b therefore supports the use of this relation for comparing thresholds across inter-pulse delays, although the absolute values should be regarded as effective thresholds in the moderate-intensity regime [32]. Figure 3b plots the squared crater diameter versus incident pulse energy, from which the ablation threshold fluence is derived via linear extrapolation to *D*^2^ = 0. Figure 3c presents the extracted threshold fluence *F* as a function of inter-pulse delay *τ*. The threshold first decreases slightly, reaches a minimum near *τ* ≈ 1 ps, then rises gradually around 3.84–7.68 ps delays. The minimum at 1 ps indicates an enhanced ablation sensitivity, which is non-monotonic and demands a mechanistic explanation based on transient material properties.

### 2.4. Ultrafast Reflectivity Dynamics

**Figure 4:**
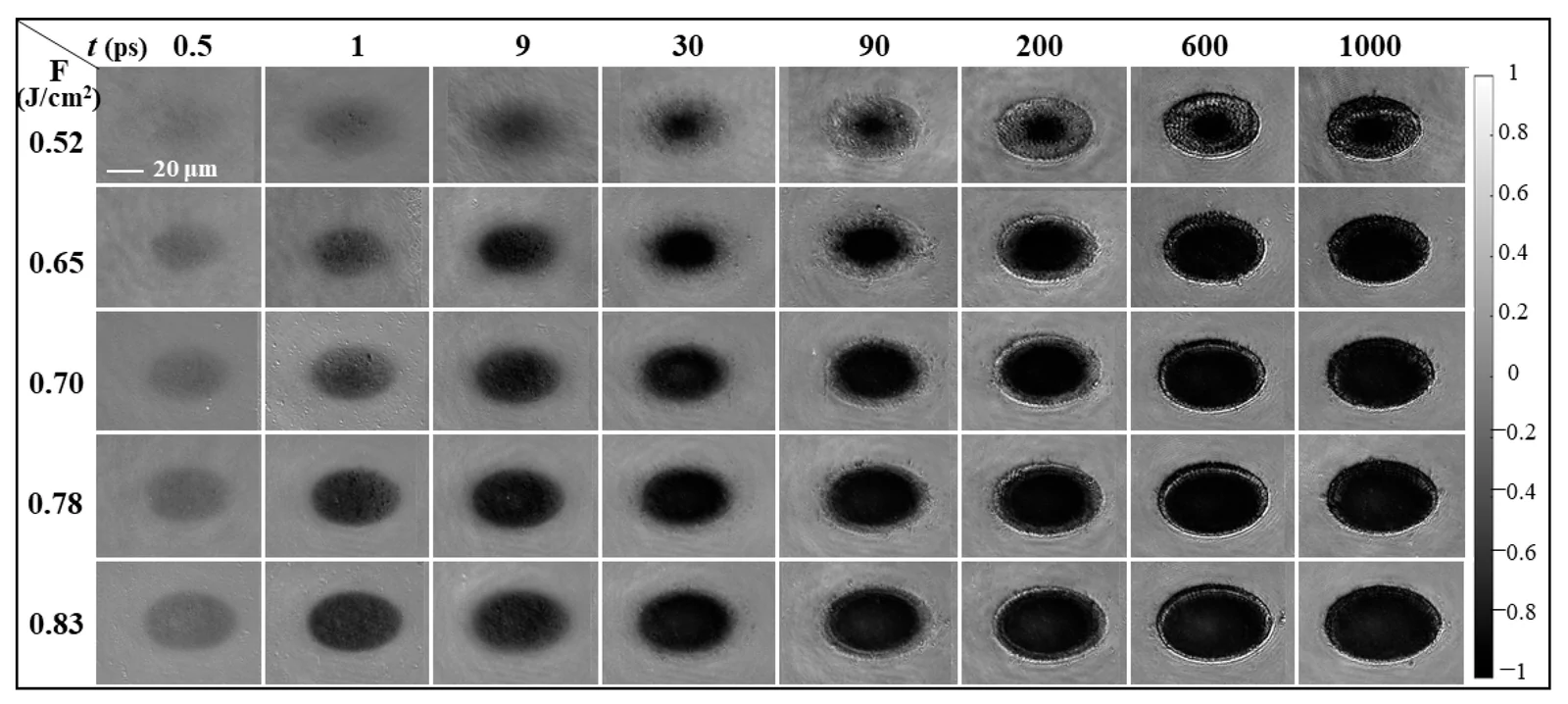
Time-resolved pump–probe reflection images of single-pulse femtosecond laser ablation of aluminum films at various pump fluences and probe delays. Each panel maps the spatial distribution of the reflected 400 nm probe intensity across the irradiated spot. At sub-picosecond delays, a uniform reflectivity drop is observed; at intermediate delays (1–100 ps), melting, thermal expansion, and acoustic relaxation produce complex contrast patterns; at delays > 100 ps, interference fringes (Newton rings) appear, indicating lift-off of the molten layer.

To uncover the origin of this threshold minimum, Figure 4 presents time-resolved pump–probe reflection images acquired at various pump fluences and probe delays. All pump fluences quoted in this section and in Figure 4 are projected surface fluences, obtained by correcting the incident fluence for the 45° angle of incidence. At sub-picosecond delays, a uniform and rapid decrease in reflectivity is observed. Comparing images acquired at different pump fluences, higher fluences (0.78–0.83 J/cm^2^) produce deeper reflectivity drops and earlier onset of interference fringes. At 0.52 J/cm^2^, the reflectivity drop develops gradually over 30–100 ps, whereas at 0.83 J/cm^2^ it is already pronounced by 9–30 ps, indicating that higher deposited energy promotes melting, thermal expansion, and acoustic relaxation, driving the material into the ablation stage at earlier times. This ultrafast drop is attributed to the electronic response characteristic of aluminum, an *sp*-band metal: for a 400 nm probe, the optical response is dominated by intraband (free-carrier) absorption [21]. Upon absorption of the 800 nm pump pulse, the free electron gas is heated via Drude absorption, raising the electron temperature T_e_ to several thousand Kelvin within ~100 fs. This creates unoccupied states below the Fermi level E_F_ and occupied states above E_F_—a phenomenon known as Fermi smearing—which enables strong intraband absorption of the 400 nm probe pulse and results in the observed reflectivity drop [12,13].

At a delay of approximately 1 ps, a clear inflection point appears in the reflectivity contrast. This inflection is consistent with the timescale for electron-phonon coupling, marking the end of the electronic heating stage and the onset of thermomechanical response (melting and thermal expansion). Subsequently, the rapid volumetric expansion of the molten layer reduces the local mass density, further modifying the optical constants and diminishing the reflectivity. It should be noted that the inflection near 1 ps may contain contributions from both the completion of electron–phonon coupling and the initial stage of thermal expansion, which partially overlap in time [33]. At longer delays (>100 ps), interference fringes (Newton rings) appear, indicating lift-off of the molten layer.

### 2.5. Quantitative Transient Reflectivity Evolution

**Figure 5:**
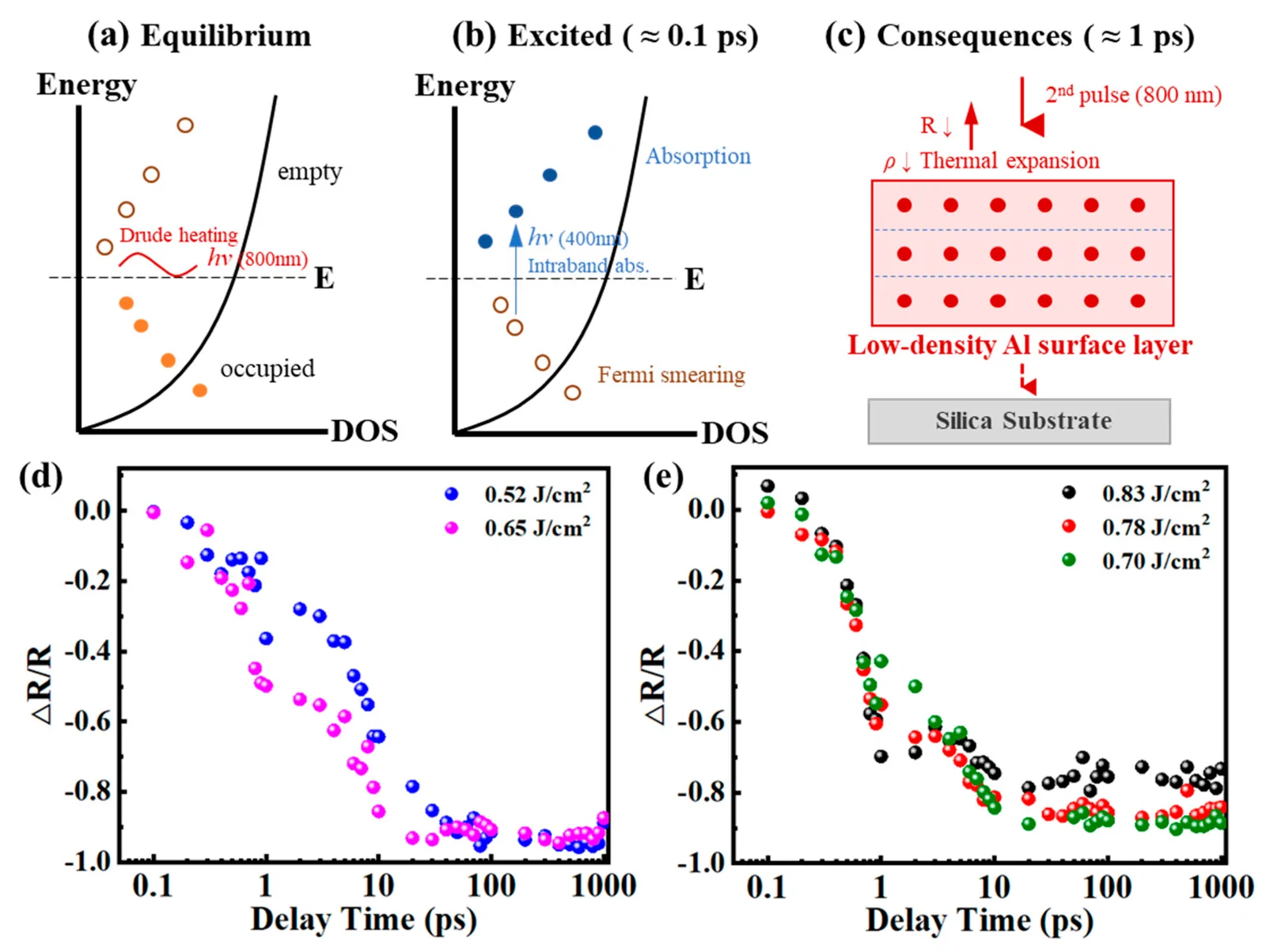
Ultrafast dynamics in Al upon femtosecond excitation. (**a**) Equilibrium: the Al sp-band density of states (solid curve) is fully occupied below the Fermi level E_F_ (dashed line) and empty above it; filled and open circles represent occupied and unoccupied electronic states, respectively. The 800 nm pump pulse heats the free electron gas via Drude (intraband) absorption. (**b**) At ≈0.1 ps, Fermi smearing creates unoccupied states (holes) below E_F_ and hot electrons above E_F_, enabling strong 400 nm intraband absorption of the probe pulse and a sharp reflectivity drop. (**c**) At ≈1 ps, thermal expansion reduces the surface density (*ρ*↓), hindering efficient energy deposition from the second pump pulse into the bulk and thereby lowering the ablation threshold. (**d**,**e**) Temporal evolution of transient reflectivity extracted from pump–probe images at different pump fluences (0.52–0.83 J/cm^2^). The reflectivity drop within 0.5 ps, the plateau at 1–10 ps, and the inflection near 1 ps are consistently observed.

Figure 5 presents both the schematic mechanism and the quantitative transient reflectivity evolution. Figure 5a–c illustrates the proposed microscopic mechanism. Figure 5a shows the equilibrium state, where the Al *sp*-band is fully occupied below the Fermi level E_F_ and empty above it. The 800 nm pump pulse heats the free electron gas via Drude (intraband) absorption. Figure 5b depicts the excited state at ~0.1 ps, where Fermi smearing creates unoccupied states (holes) below E_F_ and hot electrons above E_F_, enabling strong 400 nm intraband absorption of the probe pulse and a sharp reflectivity drop.

Figure 5c illustrates the transient surface state at ~1 ps. After electron–phonon relaxation, the surface region is in a low-density state consistent with the onset of thermal expansion. When the second pump pulse arrives at this delay, its energy is preferentially deposited within this low-density surface layer rather than being delivered deeper into the film and the substrate. This confinement of the deposited energy to the near-surface region raises the local energy density there, so that the fluence required for ablative removal of the surface material is reached at a lower incident pulse energy, which manifests as a reduction in the ablation threshold.

Figure 5d and Figure 5e present the transient reflectivity evolution at low fluences (0.52, 0.65 J/cm^2^) and high fluences (0.70, 0.78, 0.83 J/cm^2^), respectively. The grouping is intended to highlight the different thermomechanical responses: at low fluences, the reflectivity drop is more gradual and the inflection near 1 ps is less pronounced, whereas at high fluences the drop is deeper, the inflection is clearer, and interference fringes appear earlier, which reflects the varying intensity of thermomechanical response at different deposited energies. The curves confirm the ultrafast electronic drop within the first 0.5 ps, followed by a fluence-dependent plateau and a subsequent gradual decrease on the picosecond time scale. The inflection near 1 ps is consistently observed at the higher pump fluences employed in our pump–probe measurements, and its temporal position coincides with the delay at which the ablation threshold reaches its minimum in Figure 3c. This coincidence suggests that when the second pump pulse arrives at ~1 ps, the material has entered a transient state in which the optical properties and surface density differ from the initial equilibrium condition, consistent with independent time-resolved studies of aluminum melting and thermal expansion [34,35,36]. Previous study about optical pump–probe reflectivity experiments found that the broadband reflectivity transition from solid to liquid values completes within 1.5–2 ps, supporting a thermal melting mechanism [34]. Femtosecond electron diffraction measurements showed that thin-film aluminum loses long-range crystalline order within 3.5 ps under strongly driven conditions [35]. More directly relevant to surface dynamics, polarization-resolved optical interferometry was used to measure aluminum surface motion with sub-picosecond resolution and 1 nm accuracy, and found that thermal expansion is initiated within the first few picoseconds after excitation, although it is initially restrained by the native oxide layer [36]. As a result, the energy deposited by the second pulse cannot be efficiently transferred to the underlying bulk layers; instead, it is partially reflected or absorbed in the expanded, low-density surface region. This impaired energy delivery to the deeper material effectively reduces the amount of energy available for ablative removal, thereby lowering the overall ablation threshold.

## 3. Discussion

The results above reveal a coherent physical picture of the delay-dependent ablation behavior in aluminum. The ablation threshold minimum at ~1 ps originates from the transient thermomechanical state of the film: at this delay, the surface layer has undergone significant thermal expansion and density reduction following electron–phonon relaxation, while stress relaxation and plume screening, reported to dominate at longer delays, are not expected to contribute significantly at 1 ps. This low-density surface region modifies the optical properties and reduces energy coupling, preventing efficient energy deposition from the second pulse into the underlying bulk. Consequently, the energy available for ablative removal is reduced, and the apparent ablation threshold decreases. At shorter delays, the second pulse arrives before significant expansion occurs; at longer delays beyond our measurement range, previous studies report stress relaxation and plume screening dominate [20]. The observed non-monotonic threshold dependence thus reflects the competition among electronic excitation, thermal expansion, stress relaxation, and plume screening on ultrafast timescales, indicating that ablation threshold may be dynamically controlled by tuning the inter-pulse delay. The ~1 ps threshold minimum observed here occurs in a different delay regime from the threshold increase reported for aluminum at 20–200 ps [20]. In the latter work, the threshold rise was attributed to stress relaxation and plume screening, which become dominant at tens of picoseconds. At ~1 ps, by contrast, plume screening is negligible and the threshold reduction is governed by the density-driven weakening of energy coupling described above. These two regimes are consistent with a unified picture in which the double-pulse threshold first decreases at sub-picosecond to few-picosecond delays due to density reduction, and then increases at tens to hundreds of picoseconds due to stress relaxation and plume screening. In addition to these thermomechanical mechanisms, plasma effects may also contribute at longer delays. Regarding plasma effects, transient plasma formation and its interaction with the incident pulse are also expected to be weak at the ~1 ps delay studied here, since electron–ion decoupling is still in its early stage. Previous double-pulse experiments on metals have identified a plasma-shielding-free regime for delays ≤ 1 ps [37], while significant plume screening becomes dominant only at longer delays [20].

## 4. Materials and Methods

### 4.1. Experimental Setup

The dual-pulse femtosecond laser ablation and time-resolved pump–probe reflection microscopy experiments were performed using a Ti:sapphire laser system (Tsunami, Spectra-Physics, Santa Clara, CA, USA) delivering pulses with a central wavelength of 800 nm and a duration of ~50 fs. As shown in Figure 1, a Michelson interferometer configuration was employed to generate two collinear pump pulses with precisely controlled inter-pulse delay, which were incident on the sample at an angle of 45°. The inter-pulse delay was adjusted by translating one arm of the interferometer with a motorized one-dimensional linear translation stage, corresponding to a temporal resolution of approximately 6.7 fs per micrometer of path difference. The zero delay between the two pump pulses was determined by monitoring the spatial interference fringes formed when the two pulses overlap in time, with zero delay defined as the position where the fringe contrast reaches its maximum. This procedure provides a timing accuracy on the order of a few tens of femtoseconds. To eliminate polarization-induced artifacts, a wire-grid polarizer was inserted after the delay line, ensuring that both pulses shared identical linear polarization. It should be noted that the inclined-incidence configuration shown in Figure 1 was used exclusively for the time-resolved pump–probe reflectivity measurements, whereas the ablation threshold measurements by the extrapolation method were performed separately under normal incidence.

### 4.2. Sample and Ablation Procedure

The sample consisted of a 50 nm thick aluminum film deposited on a silica glass substrate by magnetron sputtering. During ablation, the sample was translated to expose fresh surface spots for each laser shot. The incident pulse energy was varied, and the ablation craters were characterized by scanning electron microscopy (SEM; VEGA GMS, TESCAN, Brno, Czech Republic).

### 4.3. Ablation Threshold Measurement

The ablation threshold fluence was determined using the extrapolation method. For each inter-pulse delay, the squared crater diameter D^2^ was plotted as a function of the incident pulse energy, and the threshold was extracted by linear extrapolation to D^2^ = 0. This procedure was repeated for delays ranging from sub-picosecond to several hundred picoseconds.

### 4.4. Time-Resolved Pump–Probe Reflection Microscopy

A separate 400 nm probe pulse was generated by second-harmonic generation in a BBO crystal and focused onto the same sample spot. The reflection of the probe pulse was collected by a microscope objective and recorded by a CCD camera, enabling time-resolved reflectivity measurements with sub-picosecond temporal resolution. The pump–probe measurements were performed at an incidence angle of 45°. The incident pulse energies delivered to the sample were E = 9.2, 11.6, 12.4, 13.9, and 14.7 μJ. Due to the oblique incidence, the circular Gaussian beam footprint is elongated into an ellipse along the incidence plane. Based on the beam geometry, the minor axis of the elliptical footprint corresponds to 2 *w* = 40 μm, where *w* is the beam waist radius, and the major axis is elongated by a factor of 1/cos45°. The projected elliptical area is therefore S = π*w*^2^/cos45° ≈ 1.78 × 10^−5^ cm^2^. The projected surface fluence was calculated as the incident pulse energy divided by this elliptical area: *F* = *E*/S, which yields values of 0.52, 0.65, 0.70, 0.78, and 0.83 J/cm^2^, as reported in Figure 4.

## 5. Conclusions

In summary, we have investigated the effect of inter-pulse delay on femtosecond laser ablation of thin aluminum films using a dual-pulse setup with precisely controlled temporal separation. The ablation threshold exhibits a non-monotonic dependence on delay, reaching a minimum at ~1 ps. Time-resolved pump–probe reflection microscopy reveals that the ultrafast reflectivity drop is dominated by intraband absorption enhanced by Fermi smearing in the *sp*-band. At delays near 1 ps, thermal expansion reduces the surface density, impeding efficient energy deposition from the second pulse into the deeper material and thereby lowering the ablation threshold. These results correlate the transient optical response with the delayed ablation threshold in femtosecond laser processing of aluminum, suggesting a route for all-optical control of ultrafast laser ablation.

## Data Availability

The authors declare that the data supporting the findings of this study are available within the article.

## References

[1] Vorobyev A.Y., Guo C. (2013). Direct femtosecond laser surface nano/microstructuring and its applications. Laser Photonics Rev..

[2] Phillips K.C., Gandhi H.H., Mazur E., Sundaram S.K. (2015). Ultrafast laser processing of materials: A review. Adv. Opt. Photonics.

[3] Guo H., Xie J., He G.Z., Zhu D.Z., Qiao M., Yan J.F., Yu J.C., Li J.Q., Zhao Y.Z., Luo M. (2024). A review of ultrafast laser micro/nano fabrication: Material processing, surface/interface controlling, and devices fabrication. Nano Res..

[4] Yang C., Ji C., Feng S., Liu Y., Wei W., Long Y. (2025). Ultrafast laser-matter interaction mechanisms and applications in functional device fabrication: Recent advances and perspectives. Appl. Phys. Rev..

[5] He G., Li J., Han H., Yan J., Xie J., Luo M., Qiao M. (2025). Exploring of the effects of thermal ablation and plasma expansion on structure formation during ultrafast laser processing of Ti6Al4V alloy. J. Mater. Process. Technol..

[6] Rethfeld B., Ivanov D.S., Garcia M.E., Anisimov S.I. (2017). Modelling ultrafast laser ablation. J. Phys. D Appl. Phys..

[7] Nedialkov N.N., Atanasov P.A., Amoruso S., Bruzzese R., Wang X. (2007). Laser ablation of metals by femtosecond pulses: Theoretical and experimental study. Appl. Surf. Sci..

[8] Gamaly E.G. (2011). Femtosecond Laser-Matter Interaction: Theory, Experiments and Applications.

[9] Jia X., Zhao X. (2019). Numerical study of material decomposition in ultrafast laser interaction with metals. Appl. Surf. Sci..

[10] Palik E.D. (1998). Handbook of Optical Constants of Solids.

[11] Ionin A.A., Kudryashov S.I., Makarov S.V., Seleznev L.V., Sinitsyn D.V. (2014). Electron dynamics and prompt ablation of aluminum surface excited by intense femtosecond laser pulse. Appl. Phys. A.

[12] Ben-Mahfoud L., Silaeva E.P., Stoian R., Colombier J.P. (2021). Structural instability of transition metals upon ultrafast laser irradiation. Phys. Rev. B.

[13] Sokolowski-Tinten K., von der Linde D. (2000). Generation of dense electron-hole plasmas in silicon. Phys. Rev. B.

[14] Ionin A.A., Kudryashov S.I., Seleznev L.V., Sinitsyn D.V., Lednev V.N., Pershin S.M. (2015). Preablation electron and lattice dynamics on the silicon surface excited by a femtosecond laser pulse. J. Exp. Theor. Phys..

[15] Wang Y., Bai H., Deng H., Zhao J., Hu J. (2026). Regulating energy deposition and nanostructure formation for superhydrophobicity with femtosecond laser dual pulses. Opt. Laser Technol..

[16] Diaz B J.C., Ivanov D., Bilmes G.M. (2023). Laser Ablation thresholds of thin Aluminium films. Ultrafast Optics 2023.

[17] Mauclair C., Mishchik K., Cheng G., Audouard E., Stoian R. (2012). Control of ultrafast laser-induced bulk nanogratings in fused silica via pulse time envelopes. Opt. Express.

[18] Tian S., He Z., Li D., Shang F., Li X., Gong H., Luo Q., Yang X. (2026). Double-pulse laser capture microdissection with a needle beam for reduced collateral damage in biological samples. Biomed. Opt. Express.

[19] Povarnitsyn M.E., Itina T.E., Khishchenko K.V., Levashov P.R. (2009). Suppression of ablation in femtosecond double-pulse experiments. Phys. Rev. Lett..

[20] Kudryashov S.I., Samokhvalov A.A., Golubev Y.D., Ivanov D.S., Garcia M.E., Veiko V.P., Rethfeld B., Mikhailovskii V.Y. (2021). Dynamic all-optical control in ultrashort double-pulse laser ablation. Appl. Surf. Sci..

[21] Kudryashov S.I., Ionin A.A. (2016). Multi-scale fluence-dependent dynamics of front-side femtosecond laser heating, melting and ablation of thin supported aluminum film. Int. J. Heat Mass Transf..

[22] Lin G., Jiang L., Ji P., Sun J., Hu J., Lian Y. (2025). Ultrafast melting, spallation, and phase explosion in femtosecond laser processing on nickel film surface investigated by atomistic simulation and transient reflectivity microscopy. Opt. Laser Technol..

[23] Wu C., Zhigilei L.V. (2014). Microscopic mechanisms of laser spallation and ablation of metal targets from large-scale molecular dynamics simulations. Appl. Phys. A.

[24] Amoruso S., Bruzzese R., Wang X., Nedialkov N.N., Atanasov P.A. (2007). Femtosecond laser ablation of nickel in vacuum. J. Phys. D Appl. Phys..

[25] Anisimov S.I., Luk’yanchuk B.S. (2002). Selected problems of laser ablation theory. Phys.-Uspekhi.

[26] Bulgakova N.M., Bulgakov A.V. (2001). Pulsed laser ablation of solids: Transition from normal vaporization to phase explosion. Appl. Phys. A.

[27] Kandyla M., Shih T., Mazur E. (2007). Femtosecond dynamics of the laser-induced solid-to-liquid phase transition in aluminum. Phys. Rev. B.

[28] Hu L., Yan D., Zou T., Xu J., Li L., Zhang R., Yang J. (2024). Ultra-corrosion-resistant aluminum alloys achieved by femtosecond laser complete remediation of microarc oxidation defects. Appl. Surf. Sci..

[29] Bezhanov S.G., Danilov P.A., Ionin A.A., Kiseleva I.V., Kudryashov S.I., Uryupin S.A., Zayarny D.A. (2018). Femtosecond laser induced nanostructuring of aluminum films of variable thickness. Laser Phys. Lett..

[30] Kwon H., Perez C., Park W., Asheghi M., Goodson K.E. (2021). Thermal Characterization of Metal–Oxide Interfaces Using Time-Domain Thermoreflectance with Nanograting Transducers. ACS Appl. Mater. Interfaces.

[31] Park H., Wang X., Nie S., Clinite R., Cao J. (2005). Direct and real-time probing of both coherent and thermal lattice motions. Solid State Commun..

[32] Zhao X., Shin Y.C. (2013). Femtosecond laser ablation of aluminum in vacuum and air at high laser intensity. Appl. Surf. Sci..

[33] Waldecker L., Bertoni R., Ernstorfer R., Vorberger J. (2016). Electron-phonon coupling and energy flow in a simple metal beyond the two-temperature approximation. Phys. Rev. X.

[34] Kandyla M. (2006). Ultrafast Dynamics of the Laser-Induced Solid-to-Liquid Phase Transition in Aluminum. Ph.D. Dissertation.

[35] Siwick B.J., Dwyer J.R., Jordan R.E., Miller R.J.D. (2003). An atomic-level view of melting using femtosecond electron diffraction. Science.

[36] Deneuville F., Chimier B., Descamps D., Dorchies F., Hulin S., Petit S., Peyrusse O., Santos J.J., Fourment C. (2013). Sub-picosecond and nanometer scale dynamics of aluminum target surface heated by ultrashort laser pulse. Appl. Phys. Lett..

[37] Semerok A., Dutouquet C. (2004). Ultrashort double pulse laser ablation of metals. Thin Solid Films.

